# Novel Frontiers in Pediatric Cardiology: Beyond the Heart

**DOI:** 10.1016/j.jacadv.2025.101697

**Published:** 2025-04-25

**Authors:** José L. López-Guillén, Pablo Pérez-López, Álvaro Solaz-García, Emma E. Williams

**Affiliations:** a Labatt Family Heart Centre, The Hospital for Sick Children, University of Toronto, Toronto, Ontario, Canada; b Faculty of Medicine, Universidad Autónoma de Madrid, Madrid, Spain; c Hospital Universitario Puerta de Hierro Majadahonda, Madrid, Spain; d Neonatal Research Unit, Health Research Institute La Fe, University and Polytechnics Hospital La Fe, Valencia, Spain; e Division of Neonatology, The Hospital for Sick Children, and Department of Pediatrics, University of Toronto, Toronto, Ontario, Canada

**Keywords:** cardiology, heart-brain axis, innovation, interdisciplinary

Cardiovascular diseases (CVDs) are considered the leading cause of morbidity and mortality worldwide.^1^ Over the last decades, physicians' efforts on counteracting this dominant trend have focused not only on increasing diagnostic and therapeutic options but also on embracing significant advances in control and preventive strategies. As the relationship between the heart and the brain receives increasing attention and the coined term “heart-brain axis” is used daily, the impact on the quality of life of neurological sequelae in cardiac patients is high. Congenital heart disease (CHD) is associated with hypoxic and ischemic alterations, and the combination of intracardiac shunting, microembolisms, and vessel wall changes, among other factors, is responsible for the development of neurological sequelae in the pediatric population.^2^ Moreover, both preoperative and postoperative brain magnetic resonance imaging studies of newborn infants with CHD have been associated with acquired brain injury, suggesting a multifactorial insult that may even start in utero. Advances in neuromonitoring and cardiac imaging, together with the development of novel neurobiomarkers, may promote better physician understanding of this complex interaction and thus enable improved outcomes for this high-risk population.

The transition era and changing prognosis of pediatric patients with CHD were mostly influenced by the introduction of cardiopulmonary bypass in the mid-1950s and deep hypothermia with circulatory arrest in the early 1970s. This practice launched the platform for intracardiac repair of most lesions. The extent to which cardiopulmonary bypass technology may alter brain perfusion in the pediatric and neonatal population remains an unclear entity. Constant remodeling of many surgical skills and new repair techniques have, however, been redefined, and the long-term survival of patients with complex congenital heart abnormalities has continued to improve.^3^ Nonetheless, recent data have highlighted the potential nervous system involvement prior to cardiac surgery and also the impact of subsequent neurological dysfunction in cardiac patients as they advance into childhood. Such findings have dramatically changed the perception of approaching and preparing for cardiac surgery, and this remains an important topic when counseling parents prenatally. The introduction of developmental care pathways for infants with CHD promotes an individualized approach to supporting the neurodevelopmental needs of such vulnerable patients.

Neuromonitoring systems have become essential in evaluating patients with CHD for surgery. The recognition of risk factors prior to cardiac surgery has enabled not only the maximization of neuroprotection and clinical management but has also served as a prognostic tool to predict later neurodevelopment. Near-infrared spectroscopy is the most widely recognized noninvasive system used for detecting changes in regional cerebral and somatic oxygenation. The inclusion of parameters, namely preoperative regional cerebral O_2_ (rScO_2_) and regional tissular O_2_ (rStO_2_), in critical heart diseases merits recognition in preventing the risk of hypoxic brain injury and maintaining stable hemodynamics before cardiac repair.^4^ Postcardiac repair, it has been suggested that routine neuromonitoring in neonates with continuous electroencephalogram may allow for screening of subclinical seizures, enabling early intervention if detected. In addition, new evidence has acknowledged that prolonged stays in intensive care units and use of cardiorespiratory oxygenation systems may detrimentally favor oxidative stress and subsequently cause functional damage to cells or tissues. Furthermore, this holds true in the neonatal CHD population, whereby prolonged duration of invasive mechanical ventilation is predictive of adverse neurodevelopmental outcomes. Therefore, novel implemented strategies to control reoxygenation should target limiting the amount of oxidative stress in this high-risk group of pediatric and neonatal patients in order to reduce morbidity and mortality during their postoperative course.

As unresolved anatomical decisions became apparent in cardiac surgery, the revolutionary introduction of 3D models allowed illustration of complex cardiovascular anatomy. Such technology rapidly expanded and currently has been utilized to create specific anatomical replicas for diagnosis and treatment planning of congenital heart anomalies. Effective replication prior to surgical procedures has been reported to aid in decision-making in those cases considered nonroutine, thus reducing intraoperative timing and providing better cardiovascular and neurological outcomes. Moreover, the introduction of four-dimensional flow magnetic resonance imaging has been extended as an additional technology focusing on comprehensive blood flow dynamics to delineate heart-brain physiological interactions. Its applications in the neonatal and pediatric population have become particularly important in tetralogy of Fallot, transposition of great arteries, pulmonary atresia, and surgical states for hypoplastic left heart syndrome to evaluate residual findings and track changes in the diameter of vessels and intracranial blood flow.^5^

Molecular strategies have also been explored in relation to cardiovascular risk assessment. The use of novel neuromarkers has provided insights into predicting long-term neurodevelopmental outcomes. Recent data on analysis of serum brain-derived neurotrophic factor (BDNF) in neonates with CHD have suggested that BDNF levels are influenced by cardiac surgery. Reperfusion injury, inflammation, and endothelial damage have been said to predispose to elevated BDNF levels in the postoperative period in patients with CHD.^6^ However, those findings should involve further investigation, as the perioperative use of prostaglandin E1 in CHD has been endorsed as a confounding factor explaining changes in blood perfusion in this population. In another study, 2 neuronal biomarkers, neuronal-specific enolase and s100B, were measured preoperatively and postoperatively in neonates with CHD. Levels of s100B increased in those with longer circulatory arrest times and were negatively associated with age at time of surgery, suggesting the potential of protective brain strategies with longer surgical wait times.^7^ Given that the determination of neurological damage prior to cardiac surgery and cardiopulmonary bypass remains unclear, the mechanisms underlying physiological interactions between the heart and the brain should also evaluate parameters such as aortic cross-clamp time, perfusion and operation time, and the need for inotropic and vasoactive support before making further assumptions. Moreover, there is a trial currently underway in France looking at the optimal timing for CHD surgery in infants with particular attention focusing on neurodevelopmental outcomes at 24 months—the results of which are highly awaited (NCT04733378).

All in all, these new approaches have reshaped the way of understanding neonatal and pediatric cardiology and will possibly lead to enhancing the cardiac and neurological well-being of future generations. A recent European survey has, however, highlighted huge variation in neuromonitoring and neuroimaging practices within the CHD population, with the development of formalized structured guidelines being much needed. From an interdisciplinary perspective, clinical management of heart diseases together with perioperative management has demonstrated evidence toward optimizing neurological development and improving cardiovascular conditions. Ensuring newborn infants and children with CHD have routine neurodevelopmental follow-up through standardized assessments is important and will contribute to clinically relevant long-term outcome data within this growing body of evidence. Only by exploring the association of pathophysiological responses between CHD and brain diseases would we better understand the impact of surgical and interventional cardiovascular techniques on the nervous system. To counteract the adverse neurological outcomes of CVDs, there is a need to unveil the 2-way dialog of heart-brain communication.

## Funding support and author disclosures

The authors have reported that they have no relationships relevant to the contents of this paper to disclose.

## References

[1] Vaduganathan M., Mensah G.A., Turco J.V., Fuster V., Roth G.A. (2022). The global burden of cardiovascular diseases and risk: a compass for future health. J Am Coll Cardiol.

[2] Sha L., Li Y., Zhang Y. (2023). Heart-brain axis: association of congenital heart abnormality and brain diseases. Front Cardiovasc Med.

[3] Sperotto F., López Guillén J.L., Milani G.P., Lava S.A.G. (2023). Advances in pediatric cardiology. Eur J Pediatr.

[4] Feldmann M., Hagmann C., de Vries L. (2023). Neuromonitoring, neuroimaging, and neurodevelopmental follow-up practices in neonatal congenital heart disease: a European survey. Pediatr Res.

[5] Yoo S.J., Hussein N., Peel B. (2021). 3D modeling and printing in congenital heart surgery: entering the stage of maturation. Front Pediatr.

[6] Fatalov K., Turan Ö., Özkan M. (2024). Pre- and postoperative levels of serum brain-derived neurotrophic factor in neonates with congenital heart defects. Turk J Pediatr.

[7] Trakas E., Domnina Y., Panigrahy A. (2017). Serum neuronal biomarkers in neonates with congenital heart disease undergoing cardiac surgery. Pediatr Neurol.

